# Evaluating Inhibitory Effects of Paclitaxel and Vitamin D_3_ Loaded Poly Lactic Glycolic Acid Co-Delivery Nanoparticles on the Breast Cancer Cell Line

**DOI:** 10.15171/apb.2020.004

**Published:** 2019-12-11

**Authors:** Sepideh Khodaverdi, Alireza Jafari, Farahnaz Movahedzadeh, Fateme Madani, Arshid Yousefi Avarvand, Siavash Falahatkar

**Affiliations:** ^1^Department of Obstetrics and Gynecology, Fellowship of Laparoscopy, Endometriosis Research Center, Iran University of Medical Sciences, Tehran, Iran.; ^2^Urology Research Center, Razi Hospital, School of Medicine, Guilan University of Medical Sciences, Rasht, Iran.; ^3^Cellular and Molecular Research Center, School of Medicine, Guilan University of Medical Sciences, Rasht, Iran.; ^4^Institute for Tuberculosis Research, College of Pharmacy, University of Illinois at Chicago, Chicago, Illinois, USA.; ^5^Department of Pharmaceutical Sciences, College of Pharmacy University of Illinois at Chicago, Chicago, Illinois, USA.; ^6^Department of Medical Nanotechnology, School of Advanced Technologies in Medicine, Tehran University of Medical Sciences, Tehran, Iran.; ^7^Ahvaz Jundishapur University of Medical Sciences, Ahvaz, Iran.

**Keywords:** Breast cancer, Anzatax, 25-Hydroxyvitamin D2, PLGA compound, Nanoparticle, *bax* genes, MCF-7 cell

## Abstract

***Purpose:*** Paclitaxel (PTX) has transpired as a significant agent in the treatment of breast cancer. Meanwhile, polylactic glycolic acid (PLGA) nanoparticles (NPs) are able to increase the anticancer effect of the PTX in the blood.

***Methods:*** Nano-precipitation was used to prepare the PLGA-PTX-VitD3 co-delivery NPs. Drug loading, encapsulation efficiency, in vitro release profile, cell viability, migration, apoptosis, and bcl2 expression of NPs were evaluated.

***Results:*** The average size of co-delivery NPs was 231 ± 46 nm. Observed was a controlled release of the PTX and vitamin D3 from co-delivery NPs between 0.5 and 240 hours. MTT showed the ability of 8 μg.mL-1 of co-delivery NPs to kill 50 % of the MCF-7; likewise, the co-delivery NPs prevented MCF-7 migration. The co-delivery NPs led 46.35 % MCF-7 to enter primary apoptosis. 60.8% of MCF-7 in the control group were able to enter the G (1) phase of the cell cycle. The co-delivery NPs increased expression of bax. In addition to its higher toxicity against MCF-7 than that of PTX, co-delivery NPs were able to release drugs continuously for a long period, which indeed increased the efficiency of the drugs.

***Conclusion:*** The effect of co-delivery NPs on MCF-7 cell viability was different from that in other drugs. In fact, the co-deliver NPs were able to release drugs continuously for a long time, this could induce primary apoptosis in the MCF-7 and decrease the metastasis and toxicity of drugs.

## Introduction

Breast cancer is among the most common neoplastic malignancies, and one of the leading causes of women’s death in the world.^1^ High instances of this malignancy is found in developed countries; the prevalence of breast cancer in Iran is lower than that of developed countries.^2^ In spite of surgery, chemotherapy and radiotherapy as therapeutic options of this malignancy, mortality rate is still high among patients with breast cancer.^2^ Currently, the co-delivery of anti-cancer drugs has been developed by assistance of biodegradables nanoparticles (NPs) such as polylactic glycolic acid (PLGA) NPs.^3-5^

PLGA NPs are one group of the commonly used polymers in manufacturing NPs thanks to their high adaptability to biological environments, ability to degrade into natural metabolites, lower toxicity and higher stability than that of liposomes and some other drug delivery systems. PLGA NPs also have the ability to modify their surface to prevent rapid absorption of this polymer by the reticuloendothelial system of the body. Toxicity and damaging the healthy tissues in the immediate area of the tumor are the result of using high doses of the drug, meanwhile, a biodegradable and biocompatible amphiphilic polymer; in fact, PLGA increases the anti-cancer effect of the drug in the blood by increasing its circulation time. In addition, studies have shown that use of PLGA in active and inactive cerebellar drugs will increase their endocytosis. Moreover, the PLGA-made NPs are of the most promising tools for transferring drugs and genes from the duct-brain.^6^ The poly vinyl alcohol (PVA) which modifies the size and distribution of small size in NPs is also one of the most widely used polymers for stabilizing NPs.^7^ Studies show that PVA is tightly connected to PLGA; the justification for this process is the hydrophobic link established between the hydroxyl functional group of PVA. The PLGA-PVA, which amongst others is one of the most successful polymers for making polymer NPs,^8,9^ could be a suitable candidate for delivery and controlled release of anti-cancer drugs such as paclitaxel (PTX).^10^

PTX is one of the new anti-cancer drugs used for many human cancers including ovarian and breast cancers. This molecule facilitates accumulation of microtubules from tubulin dimers and stabilizes them by preventing the de-polymerization of microtubules. Preventing the reorganization of the active and natural network of microtubules, this stability is essential for vital interphase and mitotic cell transplantation. In addition, PTX induces an abnormal or cluster arrangement of microtubules during the cell cycle, leading to the formation of multiple lobes of microtubules during mitosis results.^11^ PTX, in spite of its therapeutic effect, causes side effects. Short metabolized half-life and membrane transducers preventing the concentration of the drug in the cell make repeated use of relatively high doses necessary to achieve the required concentration during the appropriate time periods, which in turn leads to toxicity.^11^ However, studies have shown that combination of 1, 25 dihydroxy cholecalciferol (VitD_3_) and PTX can also enhance anti-tumor activity and accelerate apoptosis in breast cancer.^12,13^ Evidently, many studies have shown that VitD_3_ has anti-cancer effects on many types of cancer cells, it modulates the immune system and controls the paths, increases apoptosis, decreases cell growth, and regulates biological processes such as angiogenesis and the production of extracellular matrix as well.^13,14^ These effects result from the inhibition of the G1/S phase of the cell cycle and the effect on many activity regulatory of the cycling.^15^ VitD_3_ reduces the expression of anti-apoptotic proteins, and genes involved in the proliferation of uterine fibroids; and the production of extracellular matrix proteins.^16^

There is no study with respect to the anti-cancer effects of the PLGA-PTX-VitD_3_ co-delivery NPs on *bax* expression. We combined the VitD_3_ and PTX to enhance the anti-cancer function, prolonged release, reduced toxicity, increased expression of *bax* gene. The cytotoxicity of VitD_3_ and PTX loaded PLGA NPs against MCF-7 cell lines were examined by MTT method and calculated IC_50_. The apoptosis, proliferative effects of NPs and the rate of expression of the *bax* genes were also compared.

## Materials and Methods

### Cell culture

MCF-7 human breast cancer cell line was cultured in Dulbecco’s modified Eagle medium (Gibco™ DMEM) (Thermo Fisher Scientific, Waltham, MA, USA), containing 10% fetal calf serum (Gibco™ FCS) (Thermo Fisher Scientific, Waltham, MA, USA), and 1% penicillin- streptomycin (Sigma Aldrich, St. Louis, USA) 10000 units/ml at 37°C in a humidified atmosphere of 95% air and 5% CO_2_.

### Preparation of PTX and VitD_3_-loaded PLGA NPs

Nano-precipitation method was used to prepare the PLGA-PTX-VitD_3_ co-delivery NPs. The solution of PVA polymer (Thermo Fisher Scientific, Waltham, MA, USA) 1% w/v was prepared in deionized water, as an aqueous phase, under continuous magnetic stirring (FAR TEST, HPMA700) at 70°C for 5 hours to obtain a homogenous solution; likewise, 50 mg of the PLGA (Sigma Aldrich, St. Louis, USA) and 5 mg of the PTX (Sigma Aldrich, St. Louis, USA) were dissolved in 10 mL, and 5 mL of acetone (Merck, Germany), respectively. Next, 1 mL of the VitD_3_ (Sigma Aldrich, St. Louis, USA) was added to acetone (0.1 % W/V). Subsequently, the PTX was poured in the PLGA solution. Afterward, organic phase was poured in the PVA (100 mL) and stirred overnight at room temperature (around 20 ± 2°C). After the evaporation of the organic phase, the NPs were collected by centrifugation (Eppendorf, MA, USA) at 12 000 rpm for 30 minutes at 4°C and washed twice with deionized water.^17^

### Physiochemical properties of NPs

The hydrodynamic diameter and the median for NPs’ size were obtained using dynamic light scattering (DLS) and zeta potential analyzer (Malvern, UK). The morphology and diameter of NPs were screened by scanning electron microscopy (SEM) as an accelerating voltage of 20.0 kV (Philips XL-30, OH, USA) after sputtering with gold. The NPs suspensions were diluted by sterile distilled water; they were then poured onto the copper plate. After the NPs dried at room temperature, they were coated with a thin layer of gold under vacuum. The diameter of NPs was measured by digitizer software. The Fourier-transform infrared spectroscopy (FTIR)(Nicolet, Magna-IR spectrometer 550) (Thermo Fisher Scientific, Waltham, MA, USA) was used to determine the chemical groups and the interaction between the particles.^17^

### Drug loading, encapsulation efficiency of NPs

Using UV-Visible spectroscopy at the wavelengths of 228 nm and 280 nm for PTX and, VitD_3_, respectively, we could obtain standard curves (Cecil CE 7250, England). The concentration of PTX and VitD_3_ encapsulated in the NPs was determined indirectly by measuring the amount of free drug in the supernatant by UV-VIS absorption being used. The supernatant was collected by centrifugation (Eppendorf, MA, USA) at 12 000 rpm for 30 minutes at 4°C and washed twice with deionized water.^17^

The encapsulation efficiency and drug loading were calculated using the equations below:

$$\frac{Amount\;of\;drugs\;used\;to\;prepare\;NPs\;–\;amount\;of\;drugs\;in\;the\;supernatant}{Amount\;of\;drugs\;used\;to\;prepare\;NPs}\times 100$$

Encapsulation Efficiency (%):

Drug loading (%):

$$\frac{Amount\;of\;drugs\;used\;to\;prepare\;NPs\;–\;amount\;of\;drugs\;in\;the\;supernatant}{Amount\;of\;drugs\;used\;to\;prepare\;NPs\;+\;weight\;of\;PGLA}\times 100$$

### In vitro release studies

In vitro release studies of all samples were performed in PBS (PH – 7.4) containing 0.2% Tween 80. In detail, we measured 5 mg of each formulation dispersed in 1 mL of the release media. The suspension was put in a dialysis tubing (cut-off - 12.4 kDa, Cas # - D0405, Sigma Aldrich, USA) and placed in a falcon containing 50 mL of the release media. Afterwards, the falcons were placed in a shacking incubator at 37°C at 100 rpm (Labtech, S. Korea). At predetermined time intervals, falcon tubes were emptied and then replenished by fresh media. The concentration of drugs released from each sample was then determined via UV-VIS spectroscopy at the appropriate wavelengths. The kinetics of release model was Peppa-Korsmeye.^18^ A cumulative release curve of percent drug release against time was then plotted.

### Cell viability assay

MTT assay was applied to evaluate the cytotoxic effect of PLGA-PTX-VitD_3_ co-delivery NPs, PTX, PLGA-PTX and, PLGA on MCF-7 breast cancer cells.^6^ To calculate the final concentration of the PLGA-PTX-VitD_3_ co-delivery NPs, the mole scale of the NPs was converted to milligram scale and so the NPs were measured by weight balance (DORJE, KI-204, and China). Briefly, 1, 12.5, 25, 50, 100 and 200 µM of PLGA-PTX-VitD_3_ co-delivery NPs were solved into the culture medium. The cells were seeded at a density of 1.2×10^4^ cells/mL and incubated with PLGA-PTX-VitD_3_ co-delivery NPs, PTX, PLGA-PTX and, PLGA for 24 hours, respectively. Then the DMEM was changed and cells were incubated with MTT solution (Sigma Aldrich, St. Louis, USA) (0.5 mg/mL) for 4 hours. In this study, control group was treated with DMSO 0.1%.

### Migration assay

The MCF-7 cell lines were first grown in DMEM supplemented with 10% FBS to test migration assay, then seeded in 6-well plates.^19^ Next, the width of each well is scratched by sterile pipette tip (1 mm). The wells are washed by the culture medium and the picture of wounds is taken at 0 hour (NIKON, Inverted research microscope). Thereafter, medium was replaced with different concentrations of PLGA-PTX-VitD_3_ co-delivery NPs, PTX and PLGA-PTX (IC_50_ concentration) and the cells are allowed to migrate for 24 hours. After 24 hours, the picture of wound is taken from the same regions. The rate of migration (R_M_) is analyzed by measuring the distance between the scratch edges with digitizer software. The R_M_ is calculated by following equation: R_M_=(W_i_-W_f_)/t.^20^

### Cell death with DAPI staining

To evaluate cell death, the MCF-7 cell lines were grown in DMEM supplemented with 10% FBS and 1% penicillin/streptomycin exposed to PLGA-PTX-VitD_3_ co-delivery NPs (IC_50_ concentration). The MCF-7 cells were harvested by trypsin-EDTA solution, and then were centrifuged at 2000 rpm for 5 minutes. The cells were stabilized with 4% paraformaldehyde. The cells were washed by adding 500 µL of 4, 6-diamidino-2-phenylindole (DAPI) reagent and were incubated. Next, the MCF-7 cells were centrifuged and after that the buffers (Buffer RLT Plus contains guanidine thiocyanate, buffer RW1 contains a small amount of guanidine thiocyanate, and buffer RW1 Contains ethanol) were added to cell suspension. Excitation wavelength were observed at 340/380 nm by fluorescent microscopy.

### Flow cytometer and cell cycle analysis

To analyze the quantitative DNA content in MCF-7 culture cells exposed to PLGA-PTX-VitD_3_ co-delivery NPs, PTX, and PLGA-PTX, the nucleic acid stain propidium iodide (PI) was used by flow cytometry analysis. In this way, the MCF-7 cells were harvested in the DMEM and washed in PBS. The cells were fixed in cold 70% ethanol for 30 minutes at 4°C. After that, MCF-7 cells were washed with 2X in PBS and spun at 2700 rpm in a centrifuge. The MCF-7 cells were treated with ribonuclease to ensure that only the DNA, and not RNA, is stained. Next, 200 μL PI was added to stock solution. Then, to identify single cells, the forward scatter (FS) and side scatter (SS) are measured by FlowJo softwere.^21^ The PBS containing MCF-7 cell lines without any NPs was used as a control group.

### RNA extraction, cDNA preparation and real-time PCR

Total RNAs were isolated from MCF-7 cells by means of RNeasy plus mini kit )QIAGEN- Cat. No: 74134). Extracted RNA was treated with DNase I enzyme. One microgram of RNA from each sample was used for cDNA synthesis by reverse transcriptase. RNA samples were incubated at 65°C for 5 minutes with 1 μL Oligo dT (0.5 μg/μL) at a final volume of 10 μL, and then, mixed with 4 μL of 5X buffer, 0.5 μL RT Enzyme Mix Ӏ, 0.5 μL of random hexamer primer (6-mer) (100 µM) and, 4.5 μL of RNase Free dH_2_O. Finally, the 0.2 mL of prepared buffer was used to PCR reaction. After incubation of the buffer at 42°C for 15 min, and at 85°C for 5 minutes, the mixture was transcribed into cDNA. The quality of cDNA is evaluated by electrophoresis assay. 2 μL cDNA of each sample was used for real-time PCR in 16 µL reaction mixtures with 6 µL of SYBR premix ex taq and 0.4 μL of specific forward and reverse primers. The forward β-actin (as a housekeeping gene) primer (5′-GCTCAGGAGGAGCAAT-3’) and reverse β-actin primer (5′-GGCATCCACGAAACTAC-3’) also were used to normalize the target gene expression. The reverse primer sequence of *bax* primers set was 5’-CAT CTT CTT CCA GAT GGT GA-3’ and forward primer sequence was 5’-GTT TCA TCC AGG ATC GAG CAG-3’. Eventually, 0.25 μL of ROX (50X), 0.8 μL of cDNA and, 8 μL of dH_2_O are added to buffer. Initial denaturation step in real-time PCR was run at 95°C for 10 minutes. Second step was done with 40 cycles at 95°C for 15 seconds and 60°C for 1 second. The Melt Curve achieved at 95°C for 15 seconds, 60°C for 1 minutes and, 95°C for 15 seconds. The real-time PCR was performed in duplicate, and relative mRNA of each target gene was determined by using the formula 2^-ΔΔCT^ (CT, cycle threshold) where ΔCT = CT (target gene) − CT (β-actin). The comparative expression level of each target gene between different samples was calculated by 2^-ΔΔCT^. In addition, melting curves used to determine non-specific amplification.

### Statistical analysis

Statistical analysis was performed using GraphPad Prism, version 5.0. The significant difference between the experimental groups and controls was assessed by one-way ANOVA and post hoc Tukey test. The results were presented as mean ± standard deviation (SD). *P* value< 0.05 represented significant differences.

## Results and Discussion

**Figure 1:**
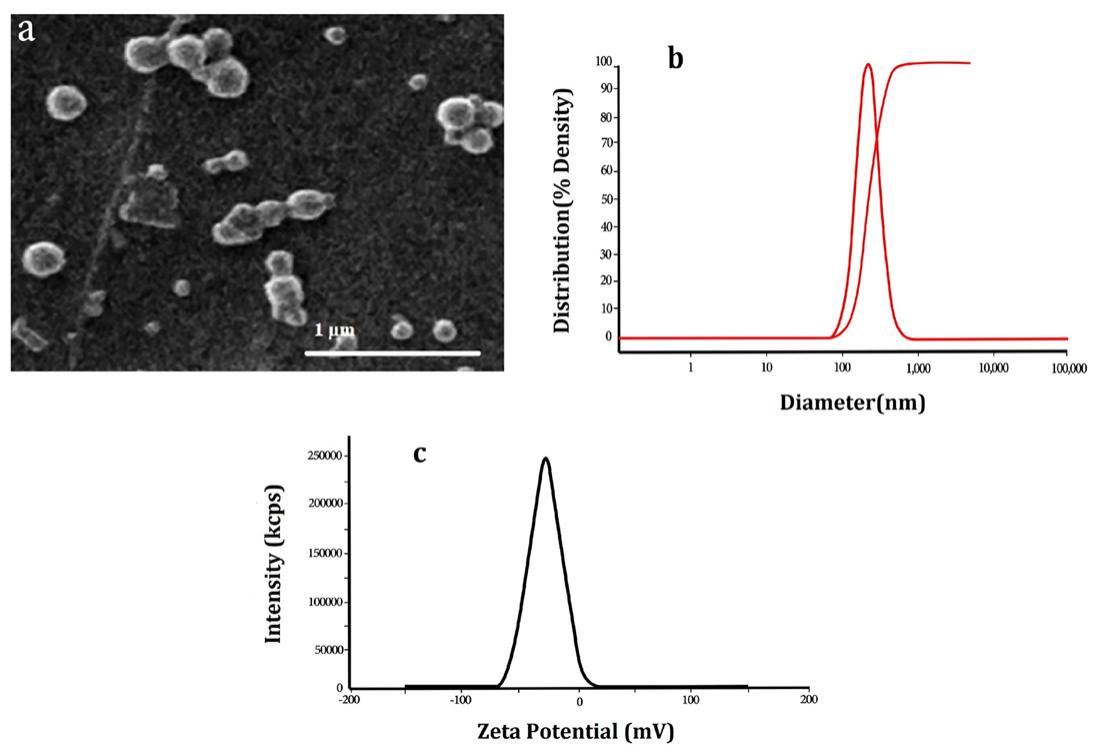
The morphology (a), the hydrodynamic diameter (b) and zeta potential of PLGA-PTX-VitD_3_ co-delivery NPs (c).

Physiochemical properties of NPs were investigated in this study are summarized in Table 1. The morphology and diameter of PLGA-PTX-VitD_3_ co-delivery NPs were done using SEM at an accelerating voltage of 20.0 KV (Philips XL-30). The diameter of PLGA-PTX-VitD_3_ co-delivery NPs measured 231 ± 46nm (Figure 1a). The hydrodynamic diameter of PLGA-PTX-VitD_3_ co-delivery NPs was measured by DLS (Stereoscope-IN A-ONE Enc., Korea) <250 nm as shown in Figure 1b. The NPs were homogeneously dispersed. The zeta potential of NPs was -20 to -10 mV, which related to the presence of polyvinyl alcohol on the surface of the PLGA NPs. The zeta potential prevents the agglomeration of particles by using electrical repulsion (Table 1).

**Table 1:** Physiochemical properties of PLGA, PLGA-PTX NPs, and PLGA-PTX- VitD_3_ co-delivery NPs

**Sample**	**Size (nm)**	**PDI**	**Zeta (mV)**	**Relative EE %**	**Relative DL (mg/g)**
PLGA NPs	60±09	0.02	-18.4	-	-
PLGA-PTX NPs	200±27	0.04	-20.3	85%	7
PLGA-PTX-VitD_3_ NPs	231±46	0.06	-15.7	PTX 82%	PTX 6
				VitD_3_ 95%	VitD_3_ 1.5

PGLA, poly lactic glycolic acid; NPs, nanoparticles; PTX, paclitaxel.

**Figure 2:**
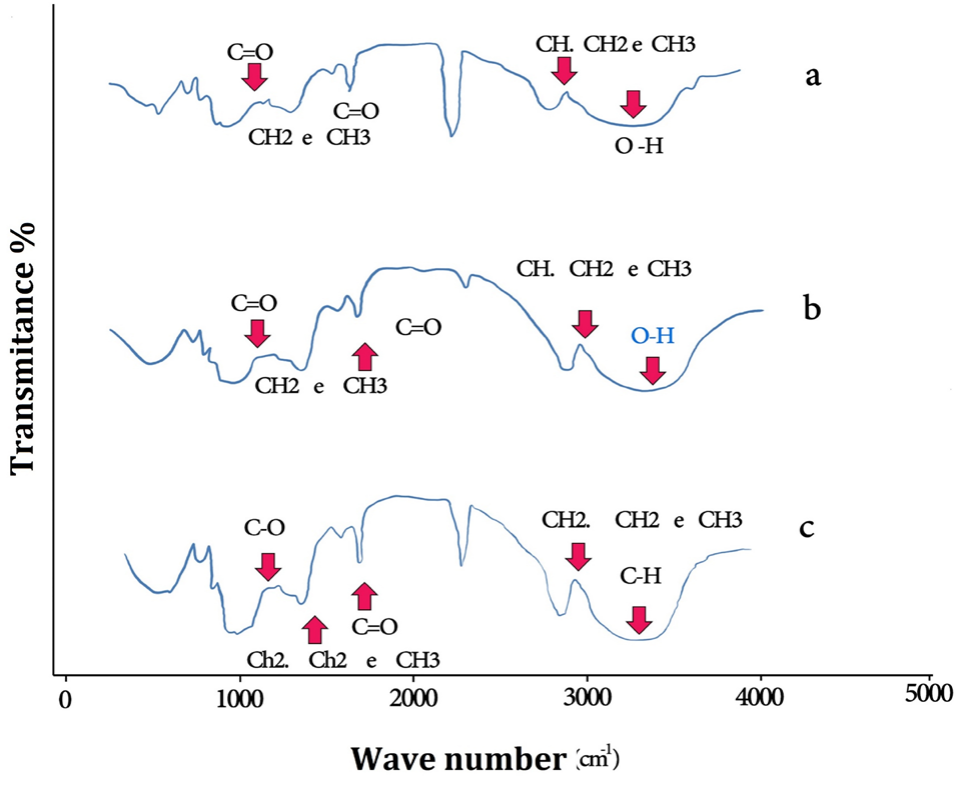
The FT-IR spectra of PLGA (a), PLGA-PTX NPs (b) and PLGA-PTX-VitD_3_ co-delivery NPs (c).

Figure 2 shows FT-IR spectra of PLGA (a), PLGA-PTX NPs (b) and PLGA-PTX- VitD_3_ co-delivery NPs (c). As shown in Figure 2a, the absorption band between 3600 cm^-1^ and 3400 cm^-1^ allocated both the ν_s_ (O-H) and ν_as_ (O-H) of hydroxyl group in PLGA NPs.^22^ The extreme absorption band between 2700 cm^-1^ and 3000 cm^-1^ allocated tensile vibration at δ (CH, CH_2_ e CH_3_). The shoulder between 1750 cm^-1^ and 1760 cm^-1^ showed a tensile vibration at δ (C=O).^23^ The absorption peaks between 1250 cm^–1^ and 1500 cm^–1^ indicated change shape ofδ (CH_2_ e CH_3_). The absorption peaks between 1150 cm^–1^ and 1300 cm^–1^were related to tensile vibration at δ (C=O).^24^ According to studies, the absorption peaks of PTX were between 3305 cm^–1^ and 3479 cm^–1^ of which were related to tensile vibration at δ (N-H). The tensile vibration at δ (C-H) is also shown between 2889 cm^–1^ and 2970 cm^–1^. The shoulder between 1703 cm^–1^ and 1732 cm^–1^ was in regard to tensile vibration at δ (C=O).^25^ In current study, the absorption peaks of PTX and vitamin D are not observed in the FTIR spectrum of PLGA-PTX NPs (Figure 2b) and PLGA-PTX-VitD_3_ co-delivery NPs (Figure 2c), which may indicate the complete encapsulation of PTX and vitamin D into the PLGA NPs.^26^

**Figure 3:**
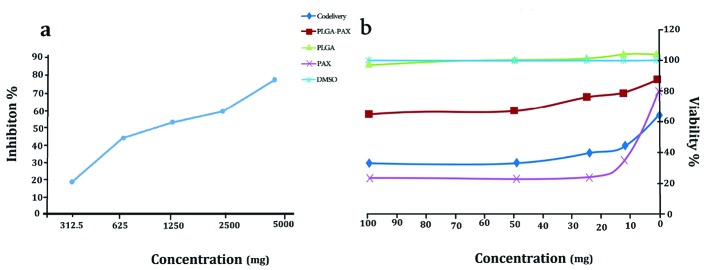
The MCF-7 cell viability in exposure the vitamin D_3_ (a), PLGA, PLGA-PTX NPs, PTX, DMSO and PLGA-PTX-VitD_3_ co-delivery NPs (b).

Cell viability assay revealed that PLGA NPs and PLGA-PTX NPs have no toxic effects on the MCF-7 cell line. As shown in Figure 3, the IC_50_ of PTX, and PLGA-PTX- VitD_3_ co-delivery NPs against MCF-7 cell lines calculated 7.5 µg/mL and 8 µg/mL, respectively (Figure 3a). VitD_3_ did not show toxic effects at concentrations of about 1000 μg/mL (Figure 3b).

Madani et al at 2017 prepared PLGA-PTX NPs via single emulsion and precipitation methods with variable parameters including drug concentration, aqueous to organic phase volume ratio, polymer concentration, sonication time and PVA concentration.^17^ Cerqueira et al at 2017 prepared the PLGA NPs embedded with PTX and coated with hyaluronic acid (HA-PTX-PLGA) to target the drug to breast cancer cells. New formulation of HA-PTX-PLGA NPs showed high potential to decrease IC_50_ of PTX.^27^ Turino et al at 2017 prepared the PLGA-NPs, coated with L-ferritin, for the simultaneous delivery of PTX into MCF-7 cells. The results confirmed that NPs decolorated with L-feritin increased PTX cytotoxicity. Moreover, the coating increased NPs stability, thus reducing the fast release of a specific drug before reaching the target. ^28^ Tran prepared dual anticancer agents such as PTX and artesunate (ART), which were loaded into PLGA NPs by solvent evaporation technique from oil-in-water emulsion, stabilized with Tween 80. The PTX and ART released from NPs in a controlled release pattern. Moreover, compared to free drugs, PTX and ART preparation increased cytotoxicity on breast cancer cell-lines.^29^

**Figure 4:**
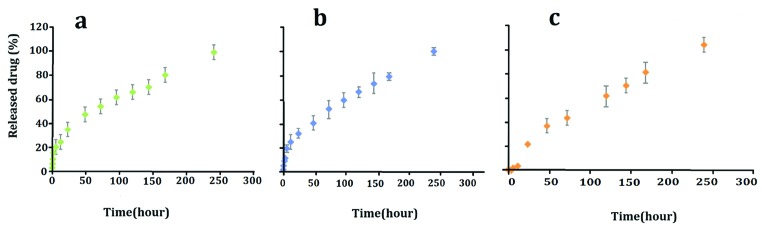
PTX release from PLGA NPs (a), PTX release from co-delivery NPs (b) and VitD_3_ release from co-delivery NPs (c).

Based on the results, there was no significant difference between the PTX release from PLGA-PTX NPs and the PTX release from PLGA-PTX-VitD_3_ co-delivery NPs between 0.5 and 240 hours (*P* > 0.05) (Table 2, Figure 4). No significant difference release of the PTX from PLGA-PTX NPs was also observed between 0.5 and 3 hours (*P* > 0.05). The release of VitD_3_ from PLGA-PTX-VitD_3_ co-delivery NPs between 0.5 and 12 hours did not also show meaningful differences (*P* > 0.05). Hence, the release of VitD_3_ from PLGA-PTX-VitD_3_ co-delivery NPs increased at 240 hours, continuously (*P*<0.05). The release of PTX and VitD_3_ from PLGA-PTX-VitD_3_ co-delivery NPs showed a noticeable difference between 3 and 96 hours (*P*< 0.05) (Table 2, Figure 4).

**Table 2:** In vitro drug release from the optimized sample. The percentage of cumulative release of PTX from PLGA-PTX, PTX from Co-delivery NPs and vitamin D from Co delivery NPs against time

**Time treatment**	**PTX from PLGA-PTX**	**PTX from Co-delivery**	**Vitamin D from Co-delivery**
0.5	2.04 ± 0.32^Ai^	3.00 ± 0.90^Ah^	0^Ah^
1	5.67 ± 0.88^Ai^	6.00 ± 1.90^Ah^	0^Ah^
2	7.67 ± 1.17^Ai^	10.73 ± 1.22^Agh^	0^Ah^
3	12.23 ± 1.74^ABhi^	16.00 ± 5.51^Afg^	0^Bh^
6	18.03 ± 2.65^Agh^	20.00 ± 8.50^Afg^	1.00 ± 0.00^Bh^
12	23.00 ± 4.48^Afg^	24.00 ± 3.51^Af^	3.00 ± 0.58^Bh^
24	30.00 ± 3.06^ABf^	35.00 ± 7.02^Ae^	19.30 ± 1.56^Bg^
48	38.00 ± 3.50^ABe^	48.00 ± 7.44^Ad^	34.00 ± 4.58^Bf^
72	49.03 ± 6.20^ABd^	54.00 ± 3.51^Ad^	40.97 ± 6.72^Bef^
96	55.97 ± 3.87^ABcd^	62.00 ± 4.04^Ac^	46.00 ± 4.51^Be^
120	60.00 ± 2.73^Ac^	66.00 ± 7.05^Ac^	57.00 ± 7.51^Ad^
144	69.00 ± 10.17^Ab^	70.00 ± 7.51^Ac^	64.97 ± 3.05^Ac^
168	74.00 ± 3.51^Ab^	80.17 ± 5.67^Ab^	75.00 ± 7.21^Ab^
240	92.00 ± 4.04^Aa^	98.00 ± 2.00^Aa^	96.00 ± 5.03^Aa^

* Values are the means ± standard error (n=3). Different capital letters (A, B, C, ...) in the same row shows significant difference among treatments for each time and different lowercase letters (a, b, c, ...) in the same column shows significant difference among different times (Tukey’s test, *P* < 0.05).

**Figure 5:**
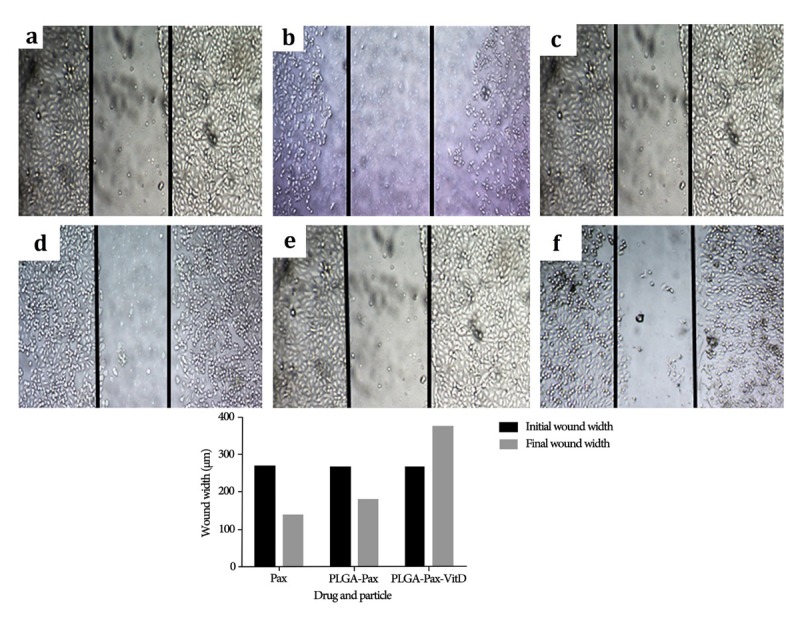
The mean of the distance scratch edges (n = 15) for PLGA-PTX- VitD_3_ co-delivery NPs (b), PLGA-PTX NPs (d), and PTX (f) at the beginning (a, c and e), and after 24 hours of experience.

Figure 5 shows the migration of MCF-7 cell line at the beginning (as a control) and after 24 hours of exposure, when exposed to PLGA-PTX-VitD_3_ NPs, PLGA-PTX NPs, and PTX. The results showed that PLGA-PTX-VitD_3_ co-delivery NPs is able to prevent metastasis of the MCF-7 cell lines.

**Figure 6:**
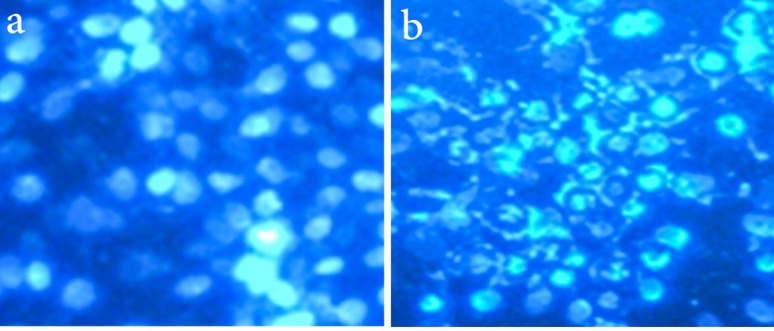
The fragmented nuclei and compact chromatin represent the cell death in the MCF-7 cell lines at present of PLGA-PTX- VitD_3_ co-delivery NPs (b), and control group (a)

As we show in Figure 6, the nucleus of MCF-7 cell lines upon exposure to PLGA-PTX-VitD_3_ co-delivery NPs become fragment and dense chromatin, which showed MCF-7 cells death.

**Figure 7:**
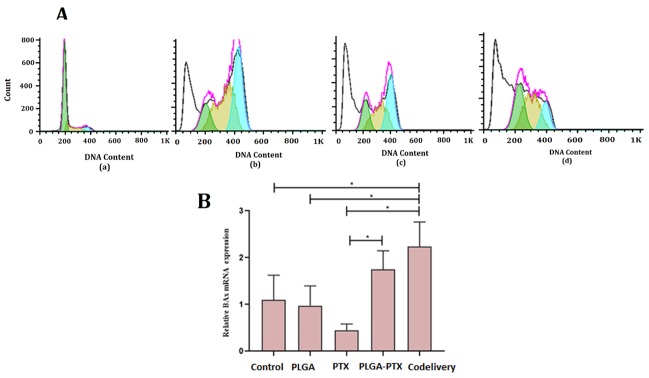
** (A)** The percentage of DNA content of MCF-7 cells in the exposure with control (a), PTX (b), PLGA-PTX (c), and PLGA-PTX- VitD_3_ co-delivery NPs (d). **(B)** Expression of *bax* gene of MCF-7 cells in the exposure with PLGA-PTX, PLGA-PTX- VitD_3_ co-delivery NPs, PTX, PLGA and control group.

Figure 7A shows the flow cytometer and cell cycle analysis of PLGA (a), PTX (b), PLGA-PTX NPs (c), PLGA-PTX-VitD_3_ co-delivery NPs (d). We found that there was a significant difference between PTX, PLGA-PTX NPs, PLGA-PTX-VitD_3_ co-delivery NPs, and the control group in the SUB G (1)/M phase (*P* = 0.030). The PLGA-PTX-VitD_3_ co-delivery NPs were able to induce early apoptosis on MCF-7 cells (41.425 ± 6.965) which showed a significant difference from the control group (11.245 ± 0.431) (*P* = 0.028). The results of this study also showed that PLGA-PTX, PTX and PLGA-PTX-VitD_3_ co-delivery NPs were able to block the MCF-7 cells at G (1)/M phase, which showed a significant difference in comparison with the control group (*P*< 0.05).

The results of this study showed that the expression of *bax* gene in the MCF-7 cells exposed to the PLGA-PTX-VitD_3_co-delivery NPs was significantly higher than the control group. Gene expression level at the PLGA-PTX-VitD_3_ co-delivery NPs showed an increase in comparison with PTX (Figure 7B). The expression of the *bax* gene in the PLGA-PTX NPs also showed a significant increase compared to free PTX (Figure 7B).

Kang et al found that anti-tumor activity of PTX from microemulsion containing PLGA was enhanced against MCF-7 cell lines.^30^ Shiny et al in 2013 prepared the PTX loaded microspheres using the blends of PLGA, poly-caprolactone. Shiny studies showed that blend of PLGA with poly-caprolactone resulted in complete release of the drug in a period of 30 days.^31^ Jin et al in 2009 also prepared the PTX-loaded PLGA NPs and determined cytotoxicity of released PTX for MCF-7 cell lines. The PTX-loaded PLGA NPs demonstrated that released PTX retained its bioactivity to block cells in G (2)/M phase.^32^ Vivek et al in 2014 investigated “smart” pH-responsive drug delivery system based on chitosan nano-carrier for its potential intelligent controlled release and enhancing chemotherapeutic efficiency of oxaliplatin.^33^ Furthermore, it was found that expression of *bax* gene was significantly up-regulated.

This study does not concentrate on anti-tumor activity of VitD_3_ (as aconfounder) loaded in the PLGA-PTX-VitD_3_ co-delivery NPs. Indeed, we did not investigate the synergistic impact of VitD_3_ and PTX. The cytotoxic effects of vitamin D and PLGA against cancer cells have been studied separately. It would not be cost-efficient to do repetitive tests.

## Conclusion

Current study showed that PTX and VitD_3_ were loaded completely into the PLGA NPs. Despite the fact that the toxicity of the PLGA-PTX-VitD_3_ co-delivery NPs against MCF-7 cell lines became lower than that of free PTX, the co-deliver NPs were able to release drugs continuously for a long time which could increase the efficiency of drugs. The PLGA-PTX-VitD_3_ co-delivery NPs not only was able to prevent the formation of metastasis, but also, they induced primary apoptosis in the MCF-7. Furthermore, we found that the PLGA-PTX-VitD_3_ co-delivery NPs increased expression of *bax* gene in the MCF-7 cell lines.

## Ethical Issues

This study was in accordance with the declaration of Helsinki; however, because we just used leftovers from primary cell line the need for ethical approval was not applicable.

## Conflict of Interest

Authors declare no conflict of interest in this study.

## Acknowledgments

We thank our colleagues from Guilan University of Medical Siences who provided insight and expertise that greatly assisted the research although they may not agree with all of the interpretations/conclusions of this paper. We would also like to show our gratitude to Dr. Bahram Soltani for sharing their pearls of wisdom with us during the course of this research, and we thank 3 “anonymous” reviewers for their so-called insights. We gratefully acknowledge the dedicated efforts of the investigators and coordinators participated in this study.
